# Factors Determining Forest Diversity and Biomass on a Tropical Volcano, Mt. Rinjani, Lombok, Indonesia

**DOI:** 10.1371/journal.pone.0067720

**Published:** 2013-07-23

**Authors:** Gbadamassi G. O. Dossa, Ekananda Paudel, Junichi Fujinuma, Haiying Yu, Wanlop Chutipong, Yuan Zhang, Sherryl Paz, Rhett D. Harrison

**Affiliations:** 1 Key Laboratory of Tropical Forest Ecology, Xishuangbanna Tropical Botanical Garden, Chinese Academy of Sciences, Menglun, Mengla, Yunnan, P.R. China; 2 University of Chinese Academy of Sciences, Beijing, China; 3 School of Resources and Environment, Northeast Agricultural University, Harbin, P.R. China; 4 Program for Field Studies in Tropical Asia, Xishuangbanna Tropical Botanical Garden, Chinese Academy of Sciences, Menglun, Mengla, Yunnan, P.R. China; 5 Key Laboratory of Biodiversity and Biogeography, Kunming Institute of Botany, Chinese Academy of Sciences, Kunming, Yunnan, China; 6 Graduate School of Environmental Science, Hokkaido University, Kita-ku Sapporo, Japan; 7 World Agroforestry Centre, East Asia Office, Kunming, Yunnan, China; 8 Conservation Ecology Program, School of Bioresources and Technology, King Mongkut's University of Technology Thonburi, Bangkok, Thailand; 9 Caraga State University, Ampayon, Butuan City, Philippines; Michigan State University, United States of America

## Abstract

Tropical volcanoes are an important but understudied ecosystem, and the relationships between plant species diversity and compositional change and elevation may differ from mountains created by uplift, because of their younger and more homogeneous soils. We sampled vegetation over an altitudinal gradient on Mt. Rinjani, Lombok, Indonesia. We modeled alpha- (plot) and beta- (among plot) diversity (Fisher's alpha), compositional change, and biomass against elevation and selected covariates. We also examined community phylogenetic structure across the elevational gradient. We recorded 902 trees and shrubs among 92 species, and 67 species of ground-cover plants. For understorey, subcanopy and canopy plants, an increase in elevation was associated with a decline in alpha-diversity, whereas data for ground-cover plants suggested a hump-shaped pattern. Elevation was consistently the most important factor in determining alpha-diversity for all components. The alpha-diversity of ground-cover vegetation was also negatively correlated with leaf area index, which suggests low light conditions in the understorey may limit diversity at lower elevations. Beta-diversity increased with elevation for ground-cover plants and declined at higher elevations for other components of the vegetation. However, statistical power was low and we could not resolve the relative importance to beta-diversity of different factors. Multivariate GLMs of variation in community composition among plots explained 67.05%, 27.63%, 18.24%, and 19.80% of the variation (deviance) for ground-cover, understorey, subcanopy and canopy plants, respectively, and demonstrated that elevation was a consistently important factor in determining community composition. Above-ground biomass showed no significant pattern with elevation and was also not significantly associated with alpha-diversity. At lower elevations communities had a random phylogenetic structure, but from 1600 m communities were phylogenetically clustered. This suggests a greater role of environmental filtering at higher elevations, and thus provides a possible explanation for the observed decline in diversity with elevation.

## Introduction

Biotic communities are differentiated in space and time, and understanding this feature of biodiversity is fundamental to ecology [1]. Despite long recognition, the patterns and mechanisms of changes in species diversity and composition with altitude and latitude remain controversial [2]. As is well known, species diversity increases from the poles to the tropics. It is also widely accepted that patterns of change in species diversity with elevation are based on parallel changes in climate with elevation, and that altitudinal gradients of species diversity in humid regions often mirror the latitudinal pattern [3], [4]. Nevertheless, although biologists have studied patterns of species diversity and compositional change along altitudinal gradients for over a century [2], [3], [5]–[11], ecologists are still without consensus on a satisfactory account for the various patterns found. Recent evidence suggests that, although diversity decreases at high elevations, the pattern of change is variable and can include a monotonic decrease in both plant and animal diversity [12], [13], a hump-shaped pattern [14], [15], or even both patterns at different sites within a given region [9]. Many factors including productivity, climatic variation, edaphic factors, and biotic factors might explain this changing pattern, but none of these is unambiguous. To what degree floristic composition is determined by environmental factors also depends on other key ecosystem processes, such as dispersal limitation and biotic interactions, and is hotly debated [16]–[18].

Altitudinal variation in floristic diversity was critically reviewed by Rahbek [2], [4]. He argued that species richness has a mid-altitude peak, and suggested that the differences among studies may be partly due to sampling regime and the influence of the size of the area sampled. To avoid this possible artifact, more spatially explicit sampling across a variety of scales is required [19]. More studies are also required that attempt to link the elevational patterns of compositional change to the factors that might induce these patterns [14].

The relationship between species diversity and biomass is also poorly understood, with different views as to whether biomass or productivity controls or is controlled by species diversity [20]–[22]. However, the relationship has been shown to be unimodal in many systems (i.e. the highest number of species is observed at intermediate levels of biomass) [23]–[25]. Few species have the physiological tolerance to survive in low productivity or high disturbance environments [25]–[27], whereas at high productivity or low disturbance sites competitive dominance may lead to the exclusion of some species. Relatively few studies have examined relationship between biomass (woody biomass), diversity, and elevation [28], [29]. The biomass pattern will depend on variation in the density of stems and canopy height along the elevational gradient. Temperature may also directly affect plant life-history strategy and hence wood density [30]. Therefore, a deeper understanding of variation in species diversity, species composition and biomass with elevation may serve to elucidate the factors affecting each of these components.

Tropical volcanoes are understudied ecosystems, although often considered hotspots of biodiversity due to the non-equilibrium states of volcanic sites [31]–[33]. Tropical volcanoes especially those forming oceanic islands, such as the famous examples of the Galapagos, Hawaiian, and Bonin archipelagos, have been important drivers of speciation. It has also been suggested that tropical volcanoes have contributed to increased rates of evolution globally as a consequence of the SO_2_ and aerosols that they released into the atmosphere, causing ozone depletion and hence increased ultra-violet radiation (a mutagenic) [34]. The environments immediately resulting from volcanic eruptions are not suitable for most life forms [35], but over time, as a soil develops, detritivore and scavenger based communities are replaced by early successional communities dominated by pioneer plants and associated fauna, and thereafter community recovery proceeds more rapidly [31], [36], [37]. However, to date most of the studies on volcanoes have dealt with the recolonisation and successional processes following eruption [31], [38], [39]. Tropical volcanoes comprise an important ecosystem, particularly in SE Asia, that differs in important ways from non-volcanic mountain ecosystems in the tropics. Whether the biotic processes that structure elevational change in species diversity, community composition and biomass are similar to those on mountains derived from uplift is not well understood. Importantly, being younger and derived from ash and cinder over large areas, volcanic soils may be less spatially variable, at least under similar climates [40], and thus variation in below-ground processes is likely to be less important in structuring variation in the above-ground biotic community. In contrast, on non-volcanic mountains vegetation transitions are often associated with a marked change in soil properties [41]. Thus, volcanoes may provide a window on the mechanisms driving elevational changes in plant communities.

We investigated the distribution of plant diversity, composition and biomass along an elevational gradient on a tropical volcano in Indonesia. We sampled plant communities along the elevational gradient using a spatially explicit sampling protocol that enabled us to assess change both within and among stations at different altitudes. Specifically we addressed the following questions. 1) How does the floristic composition and diversity of ground-cover, understorey (2 cm≤dbh<10 cm), subcanopy (10 cm≤dbh<30 cm) and canopy (dbh≥30 cm) vegetation change with elevation. 2) How are these patterns related to variation in other factors, including slope, tree basal area, leaf area index, canopy openness, canopy height, and tree density. And, 3) how does above ground biomass change in relation to elevation and diversity. We also investigated how the phylogenetic pattern in plant assemblages varied with elevation, as this can shed light on the species assembly processes [42].

## Methods

### Study site

**Figure 1:**
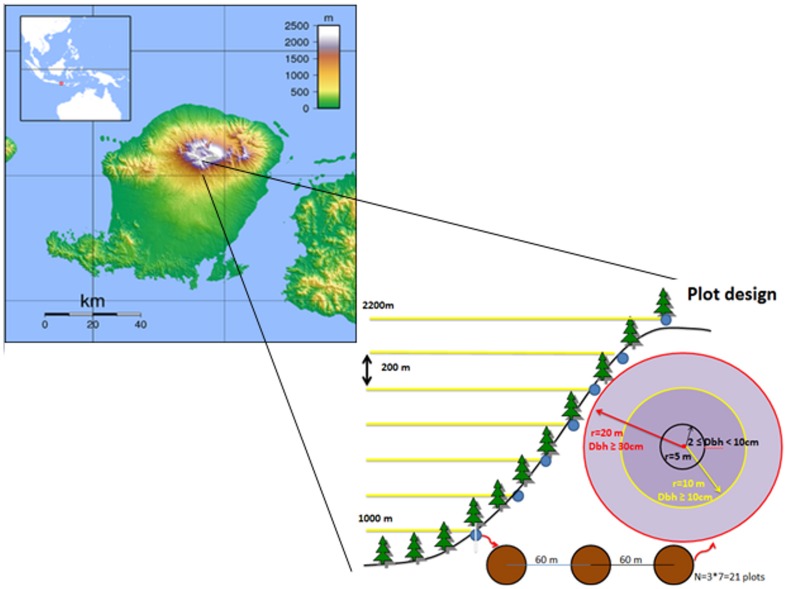
Map of the Island of Lombok and Mt. Rinjani, the study site, and the sample design. It was not possible to sample vegetation below 1000 m because of human disturbance and above 2000 m were fire-maintained grasslands. Access was only possible through using the main hiking trail on the north slope of the mountain.

We conducted our research at Rinjani National Park (116°18′–116°32′E; 8°18′–8°33′S; altitude 550–3726 m asl), Lombok, Indonesia (Fig. 1) in August 2010. The park receives average rainfall 2000 mm per year and average daily maximum temperatures range from 23° to 30°C at 550 m asl. The forest has been classified into three vegetation zones according to elevation: lower montane (600–1500 m asl), pre-montane (1500–2000 m asl) and montane (2000–2600 m asl). Because Rinjani lies within the major transition zone of Wallacea its flora and fauna mark a dramatic transition from SE Asian species into those which are typical of Australia and New Guinea. The park according to FAO (1981 cited by [43]) consists of 40% primary forests, 40% of savannah forest and 10% of planted forest. It protects several endangered plants, *Pterospermum javanicum*, *Swietenia macrophylla*, *Ficus superba*, and *Toona sureni*, and animals, *Presbytis* sp., *Philemon buceroides*, and *Lichmera lombokia*. Our studies were confined to natural primary forests on the northern slope of the mountain.

Rinjani's caldera forming eruption has been dated to 1257. Although Rinjani remains an active volcano recent eruptions have been confined to the inner caldera area. On the slopes of the volcano mature rain forest is evident at lower elevations and the forests on the northern slope investigated in this study may be described as being climax vegetation.

Permission to conduct this study as a part of the Fieldcourse on Biodiversity, Conservation and Sustainable Development was provided by Rinjani National Park authority. We did not collect endangered plants or any animals.

### Plant sampling

Three plots were sampled at each of seven different elevational stations located at 200 m vertical intervals from 1000 to 2200 m asl (n = 21). The national park starts at approximately 800 m elevation but below 1000 m human disturbance has affected the forest. At and above 2200 m was a fire-maintained grassland. Using the main trekking trail up the mountain from 1000 m asl, elevational stations were marked using a barometric altimeter. Plots at the same elevation were located 60 m apart along a horizontal transect running east from the main trail with the first point located 40 m off the trail. The location of sampling plots was constrained by access, which was only possibly via the main hiking trail, because of restrictions in walking off the main trail within the national park. The effect of the trail on vegetation attenuated within 1–2 m of the edge of the trail (RH, *personal observations*).

We sampled ground-cover plants (including pteridophytes, tree seedlings, grasses and climbers), understorey plants (including tree saplings, shrubs, and climbers), subcanopy and canopy trees by using nested circular plots of varying radius. For the ground-cover plants we sampled a 1 m radius circular plot. We visually estimated ground-coverage, using the following seven point scale: 6: 75–100%; 5: 50–74%; 4: 25–49%; 3: 5–24%; 2: 1–4%; 1: <1%, 0: not present. For understorey vegetation we included all plants ≥2 cm and <10 cm diameter-at-breast-height (dbh) within a 5 m radius plot. For subcanopy plants, all trees with ≥10 cm and <30 cm dbh were measured within a 10 m radius plot. Canopy plants included all trees with dbh≥30 cm within a 20 m radius plot. DBH was measured for all stems using a dbh-tape. The height of the tallest tree in every plot was determined using a clinometer. Data from the 1 m, 5 m, 10 m, and 20 m radius plots were analyzed separately.

Trees species were identified to morphospecies in the field, using a pair of binoculars to assist observation, and at least one sample of each species from each elevation station was collected for later identification. Material was identified both in the herbarium of the Bali Botanic Garden, Bedugul and at the Herbarium Bogorensis. Voucher of specimens are available at the Herbarium Bogorensis under file classification number of 951/IPH.1.02/If.8/V/2011.

### Environmental parameters

We measured the slope from the center of each plot to a point 5 m down slope using a clinometer. For some plots where the slope changed substantially within 20 m radius of the center point, we took 3–5 measurements on a line through the middle from the top to bottom of the plot and averaged the slope for the plot.

We determined leaf area index (LAI) and canopy openness by taking three hemispherical photographs of the canopy from a point 50 cm above the center of each plot. Digital photographs were taken with a Nikon D70 digital camera with a hemispherical circular fisheye lens, using the automatic exposure and bracketing functions (Sigma DC HSM 5.4 mm 1∶2.8). Hemispherical photographs were imported into Gap Light Analyzer software [44] where LAI and canopy openness were calculated for each image.

### Data analysis

First, we assessed the correlation between elevation and the selected covariates, including forest structural variables, such as leaf area index (LAI), tree basal area, and canopy height. For some variables which did not show a linear trend, we fitted a nonlinear polynomial regression up to the second term. When the polynomial model fitted data better than the linear model (AIC was lower), we also tested for a hump- (or U-) shaped relationship using Mitchell-Olds & Shaw test (function *MOStest*, package *vegan*) [45], which examines whether confidence interval for the location of the hump or U lie within the range of the data.

Next, we modeled species diversity (Fisher's alpha) change in relation to elevation and slope (topographic variables) and LAI. Other components of forest structure were omitted to avoid problems of colinearity. We modeled species diversity using an information-theoretic approach. We generated a set of possible candidate models starting from the maximal model, including the variables (slope, LAI, elevation) and all possible interactive terms. Then we progressively removed components from the maximal model, respecting the principle of marginality, until we arrived at the null model. Next, we compared the likelihood of each model against all others using the function *model.avg* (*MuMIn* package in R, [46]) to provide a wAIC and, using a subset of models for which the *w*AIC is >10% of the maximum *w*AIC, we summed the *w*AIC values for the models with a particular parameter to provide the relative importance of that parameter (function *model.avg*). In order to account for possible nonlinear relationships with elevation, we first examined whether the maximal model with a linear or polynomial relationship for elevation gave the lowest AIC and then selected this model for the model averaging procedure. For species diversity, we partitioned diversity into alpha (plot) diversity and beta (among plot within elevational station) diversity, and modeled both of these separately. Beta-diversity was calculated as elevation station diversity, that is the Fisher's alpha for data pooled across all three plots from one elevational station, minus the mean plot-level Fisher's alpha at that elevation [47] cited by [48].

For compositional change among elevations stations we employed multivariate regression to partition the variance (deviance) in the species×site (plot) table. Since distance based multivariate analyses (e.g. canonical correspondence analysis (CCA) and related methods) most of the time violate the mean- variance relationship assumption, we opted to use multivariate generalized linear models (GLM) (function *manyglm* in the *mvabund* package [49] in R) to model variance in species composition among elevation stations as a function of elevation, slope, and LAI. We conducted an analysis of deviance (*anova.manyglm*) to examine which variables significantly explained the variation found in the plants community along the elevational gradient. We also calculated the percent (%) deviance explained by the model and by each variable within the model.

For above ground biomass estimation, the allometric model developed by Chave et al. [50] for the moist forest stands was selected because the site's mean annual precipitation (MAP) is between 1500 and 3000 mm [51]:

(1) AGB_est._ = 0.0509*ρ*dbh^2^*H [50].

Where dbh is diameter at breast height, H is the total height and ρ is density of the tree species.

We estimated tree height using the general simplified allometric equation between height (H) and dbh:

(2) H = c(dbh)^2/3^ [52].

We used our field measurements of the tallest tree in each plot to estimate *c*, using the mean across the three plots at each elevation. It was important for us to include a height parameter in the biomass model, because canopy height declines with elevation [53]. In order to account for the biomass for both subcanopy and canopy plants we merged the data from 10 m and 20 m radius plots, by extrapolating the data for subcanopy plants over the whole area. We used the global data base of wood density (ρ) available at http://datadryad.org/handle/10255/dryad.235 to assign wood density. We obtained wood density data for 22 species at a species-level and 22 species at a generic-level. For 13 species having no available density data, we assigned 0.56 g.cm^−3^ as the average wood density of the known wood density data from our site.

We generated a phylogenetic tree for the species pool using the online version of phylomatic (version 2), assessed the nodes and branch lengths in phylocom (version 4.2 using the function *bladj*), and conducted the phylogenetic analysis in R with the *picante* package (function *cophenetic*) by computing the mean phylogenetic distance (MPD) with the function *ses.mpd* and the net relatedness index (NRI) [42], [54] under the independent swap null model [55]. This is a null model that after randomly shuffling the species pool, draws expected communties with the same number of species as in the observed communities, while keeping the occupancy rates fixed. NRI negative and positive values are interpreted as overdispersed and clustered phylogenic structure, respectively. When NRI does not differ significantly from zero, this indicates a random phylogenetic structure [42], [54].

All analyses were conducted in R v 2.15.1 [56], using the *vegan* [57], *mvabund* [49], *MuMIn* [46] and *picante* [58] packages.

For all tests, we conducted two separate analyses, one including all stations and one including only the stations up to 2000 m (i.e omitting the station at 2200 m). This was because there was a major transition in the vegetation from forest to fire-maintained grasslands above 2000 m. For most analyses, results were qualitatively similar in both analyses. Unless otherwise stated we quote the results for the analysis including only the forest habitat (i.e. up to 2000 m).

## Results

**Figure 2:**
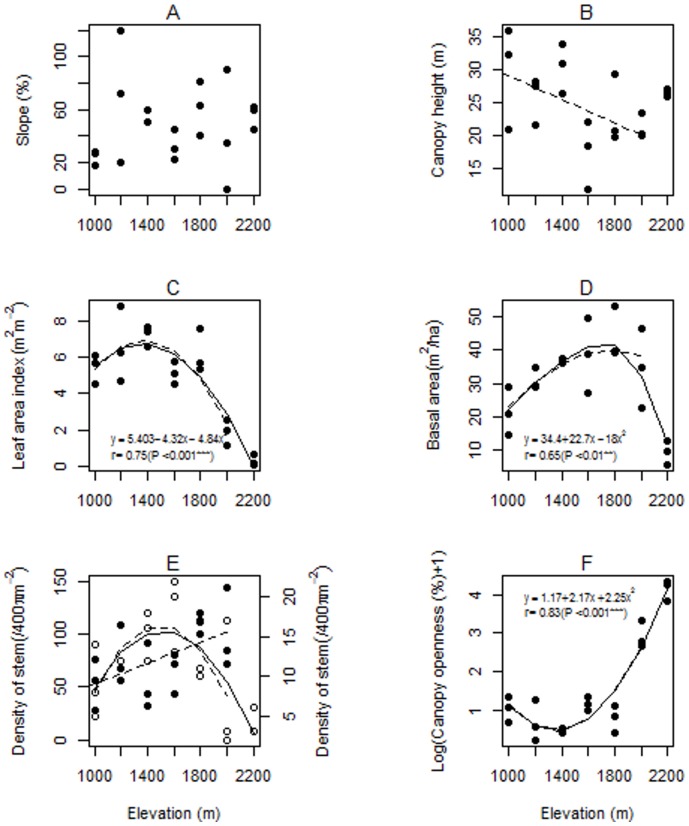
Relationships between elevation and slope, and between elevation and various components of forest structure on Mount Rinjani, Indonesia. A) slope, B) canopy height (y = 38.19+0.01x, R^2^ = 0.21), C) LAI, D) basal area, E) tree density, where filled circles are for subcanopy trees (y-axis on left, y = 3.68+0.05x, R^2^ = 0.25) and open circles are for canopy trees (y-axis on right, y = 12.39-0.48x-14.62x^2^, R^2^ = 0.33), and F) canopy openness. Solid lines indicate relationships including all elevation stations and the dashed lines indicate the relationships when the station at 2200 m (fire-maintained grassland) was omitted. Only significant (*p*<0.05) relationships are shown. The non-zero woody biomass and canopy height for the grassland station (2200 m) are because there were small numbers of isolated trees.

We recorded 902 trees and shrubs among 92 species (56 species of understorey plants, 57 species of subcanopy, 57 species of canopy plants, and 21 species shared by these three strata) and 67 species of ground-cover plants from seven elevation stations (1000 m–2200 m, 200 intervals). There was no significant association between elevation and slope (Fig. 2). However, canopy height (negatively, r = −0.50, *P* = 0.03) and subcanopy tree density (positively, r = 0.54, *P* = 0.02) were linearly correlated with elevation, while for canopy tree density (R^2^ = 0.33, *F_2, 15_* = 5.23, *P* = 0.02), tree basal area (R^2^ = 0.42, *F_2, 15_* = 7.07, *P* = 0.006), and leaf area index (LAI: R^2^ = 0.56, *F_2, 15_* = 11.95, *P*<0.001) the data suggested a humped-shaped relationships and for canopy openness (R^2^ = 0.69, *F_2, 15_* = 20.22, *P*<0.001) the data suggested a U-shaped relationship with elevation (Fig. 2). However, according to the Mitchell-Olds & Shaw test none of the polynomial relationships were significantly hump-shaped or U-shaped, although for LAI the relationship was marginally significant (F = 4.47, *p* = 0.0517).

### Species diversity

**Figure 3:**
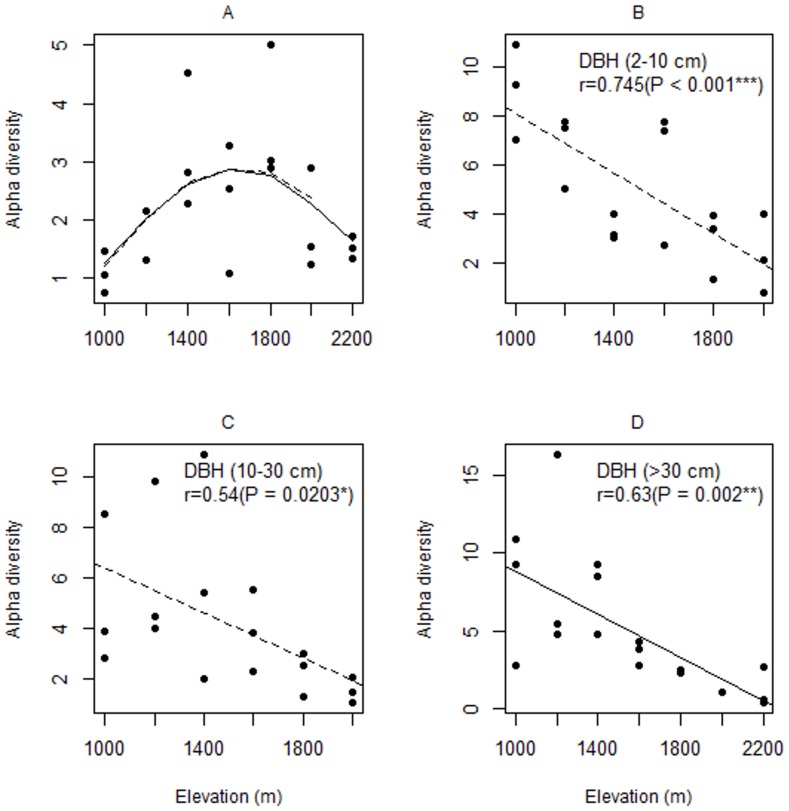
Relationships between elevation and alpha-diversity (Fisher's alpha) for different components of vegetation on Mt Rinjani, Indonesia. (A) ground-cover plants (y = 2.33+1.75x-2.39x^2^), (B) understorey plants (y = 14.24-0.006x), (C) subcanopy plants (y = 10.90-0.005x), (D) canopy plants (y = 16.43-0.007x). Solid lines indicate relationships including all elevation stations and the dashed lines indicate the relationships when the station at 2200 m (fire-maintained grassland) was omitted. Only relationships that were significant (p<0.05) in univariate regression models are shown.

Alpha-diversity (Fisher's alpha) declined with elevation for understorey plants (β = −0.007, confidence interval(CI) = −0.011—0.003), subcanopy (β = −0.006, CI = −0.012—−0.002) and canopy plants (β = −0.007, CI = −0.016—0.002), and appears to show a hump-shaped pattern for ground-cover plants (β_elevation_ = 4.06, CI = 1.80—6.32, β_elevation_^2^ = 0.73, CI = −1.80—3.25) (Fig. 3, Table S1). However, for the latter the Mitchell-Olds & Shaw test was not significant. Model averaging indicated that elevation was the only important factor in determining species diversity for understorey, subcanopy, and canopy plants, while elevation and LAI were both important for ground-cover vegetation (Table 1).

**Table 1:** Summary of the relative importance of factors in determining alpha- and beta-diversity (Fisher's alpha) of vegetation along an elevational gradient on Mt Rinjani, Indonesia.

Variables and interactive terms	alpha-diversity				beta-diversity			
	Gr.	Un.	Su.	Ca.	Gr.	Un.	Su.	Ca.
Slope	0.16*	0.18	0.24	0.25	1.00	1.00	**1.00**	1.00
LAI	**0.99** *	0.46	0.25	0.24	1.00	1.00	**1.00**	1.00
[Elevation,(Elevation)^2^]	0.99*	**1.00**	0.85	0.88	1.00	1.00	**1.00**	1.00
Elevation:Slope	0.00*	0.02	0.03	0.06	1.00	1.00	0.45	1.00
Elevation:LAI	0.01*	0.05	0.06	0.02	0.97	1.00	**1.00**	1.00
LAI:Slope	0.01*	0.01	0.01	**0.08**	0.10	0.00	0.55	0.00
Elevation:LAI:Slope	0.00*	0.00	0.00	0.00	0.03	0.00	0.00	0.00

Values were derived through a model averaging approach (*model.avg* function in MuMIn package). The variables included were slope, leaf area index and elevation and their interactive terms (2200 m station not included). For elevation we examined whether a linear or polynomial expression best fitted our data. Gr = ground-cover plants, Un = understory plants, Su = subcanopy plants, and Ca = canopy plants. Factors with parameters values that were significantly different from zero (p<0.05) are highlighted in bold.

* denotes that the polynomial relationship of elevation was selected; anywhere else only linear relationship was selected.

**Figure 4:**
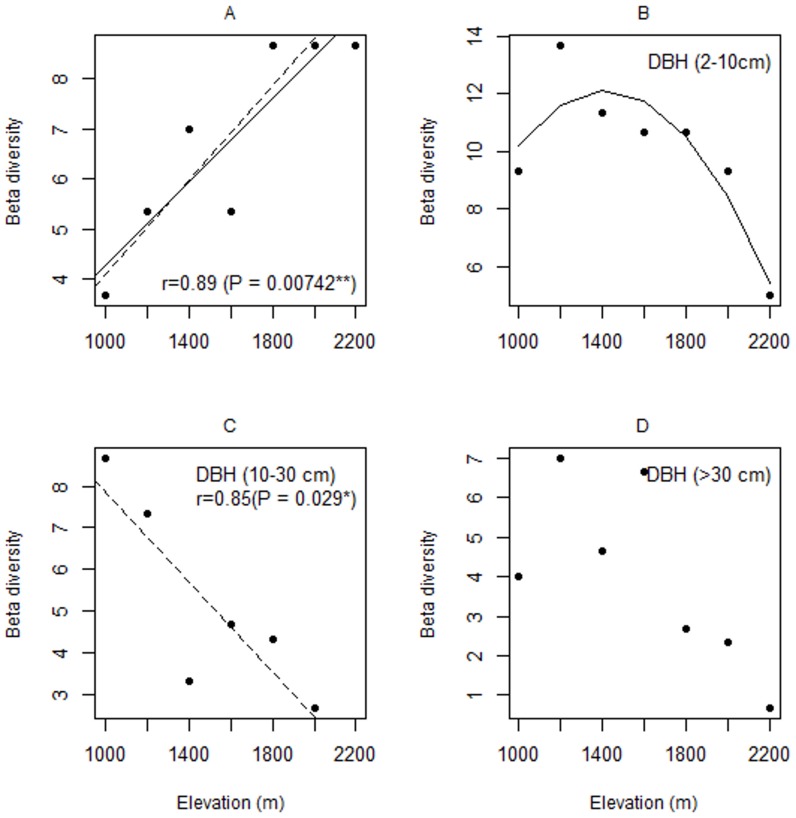
Relationships between elevation and beta-diversity (among plots with elevation stations) for different components of vegetation on Mount Rinjani, Indonesia. (A) ground-cover plants (y = 6.92+2.06x), (B) understorey plants (y = 10-4.2x-4.04x^2^, all stations 2200 m included), (C) subcanopy plants (y = 4.62-2.32x), (D) canopy plants. Solid lines indicate relationships including all elevation stations and the dashed lines indicate the relationships when the station at 2200 m (fire-maintained grassland) was omitted. Only relationships that were significant (*p*<0.05) in univariate regression models are shown.

For beta-diversity, we found a decline in diversity of understorey plants, subcanopy plants, and canopy plants, but the relationship was only significant for subcanopy plants (β = −0.03, CI = −0.033—−0.028, Fig. 4, Table 1, Table S1). Our model averaging indicated a poor capacity to differentiate among the factors (Table 1). Relative importance was high and similar among all the main effects (elevation, slope, LAI) and also both two way interactions with elevation.

### Community composition

Our multivariate GLMs for community composition among plots explained 67.05%, 27.63%, 18.24%, and 19.80% of the variation (deviance) for ground-cover, understorey, subcanopy and canopy plants, respectively (Table 2, Table S2, Fig. S1–4). For ground-cover, forest structure, slope and elevation were all important drivers of the compositional change. For understory plants and subcanopy plants, only elevation contributed significantly to the compositional change, while for canopy plants, slope and elevation were both important.

**Table 2:** Effects table for multivariate GLM analyses (function *manyglm*; resampling = “pit-trap”) of variation in community composition (species×site table) for ground-cover plants, understorey, subcanopy and canopy trees along an elevational gradient on Mt Rinjani, Indonesia.

Forest stratum	Anova test		% explained deviance	
	Variables	P-values	within model	by the model
ground-cover plants	Structure variables	0.003	61.70	67.05
	Slope	0.001	18.36	
	Elevation	0.001	19.94	
Understorey plants	Elevation	0.002	100	27.63
Subcanopy plants	Elevation	0.042	100	18.24
Canopy plants	Slope	0.22	81.76	19.80
	Elevation	0.001	18.24	

Forest structure variables included were basal area, canopy height and leaf area index (2200 m station not included).

### Above ground biomass

**Figure 5:**
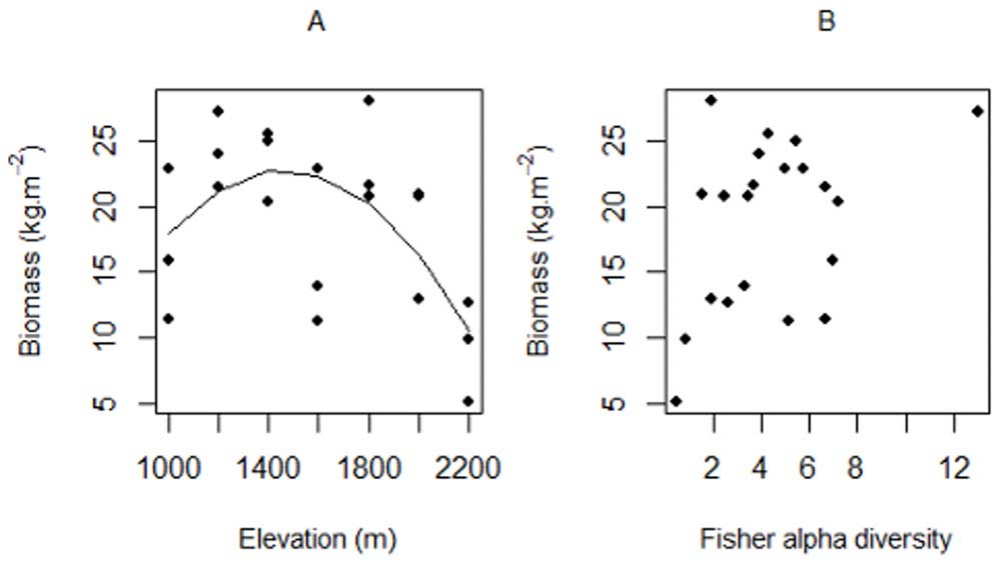
Relationships between above ground biomass and (A) elevation (y = 18.81-11.35x-14.32 x^2^, R^2^ = 0.35, all stations 2200 m included), and (B) diversity (fisher's alpha) of subcanopy and canopy vegetation combined on Mount Rinjani, Indonesia. Solid lines indicate relationships including all elevation stations and the dashed lines indicate relationships when the station at 2200 m (fire-maintained grassland) was omitted. Only significant (*p*<0.05) relationships are shown.

The highest above ground biomass was 24.24 kg.m^−2^ at 1200 m and the lowest was 9.22 kg.m^−2^ at 2200 m (Fig. 5A). There was no significant relationship between biomass and elevation when only stations up to 2000 m were included (*F_2,15_* = 0.62, *P* = 0.55). However, when the grassland station at 2200 m station was included there was a significant polynomial relationship (R^2^ = 0.35, *F*_2,18_ = 6.27, *P* = 0.55; β_1_ = −11.35±5.16, *t* = −2.2, *P* = 0.04, β_2_ = −14.32±5.16, *t* = 2.78, *P* = 0.01; Table S3), although again the Mitchell-Olds & Shaw test was not significant.

No correlation between above ground biomass and diversity (Fisher's alpha) was detected (Fig. 5B).

### Community phylogeny structure

We found that assemblages at 1600 m, 1800 m and 2200 m asl were significantly phylogenetically clustered and the assemblage at 2000 m asl was marginally significantly clustered (P = 0.07) (Table 3). Thus, communities at higher elevations showed a tendency towards phylogenetic clustering. Below 1600 m asl communities were random with respect to phylogeny (Table 2).

**Table 3:** Phylogenetic community structure among vegetation plots along an elevational gradient on Mount Rinjani, Indonesia.

Elevation (m)	No spp	MPD observed	MPD expected	NRI	P-values
1000	23	442.17	437.58±17.66	−0.26	0.61
1200	31	482.24	439.39±14.52	−2.95	1.00
1400	20	454.71	437.83±19.14	−0.88	0.80
1600	20	404.53	437.88±19.17	1.74	0.04
1800	20	401.75	438.65±19.49	1.89	0.03
2000	14	401.86	437.12±23.72	1.49	0.07
2200	3	276.25	437.42±72.82	2.21	0.02

(MPD = mean phylogenetic distance (observed and expected±standard deviation); NRI = net relatedness index).

## Discussion

### Species diversity

Alpha-diversity (plot diversity) declined with elevation for all three components of the woody vegetation (understorey, subcanopy and canopy) (Fig. 3). Beta-diversity (among plot diversity) also declined with elevation for understorey plants, subcanopy plants, and canopy plants, although the relationship was only significant for subcanopy plants. In part, this would appear to be due to the low power of the analysis for beta-diversity (Table 1, Fig. 4). Nevertheless, combined these results indicate a decline in the diversity of woody vegetation with elevation over the range of elevation covered in this study. In contrast, the alpha-diversity of ground-cover vegetation appears to follow a hump-shaped relationship, although the 95% confidence limits for the location of the maximum were outside the range of our data.

Our results corroborate the monotonic decrease in species diversity of woody vegetation with the elevation suggested from earlier studies on tropical mountains [3], [6], [41], [53], and is the expected pattern if physiological constraints, such as temperature and water stress, are the sole limiting factors. The results for ground-cover vegetation are also instructional as they indicate how other observed patterns may result from the effects of elevation on forest structure. In a tropical lowland rain forest only approximately 2% of the photosynthetically active radiation reaches the forest floor [59]. Light is therefore a strongly limiting factor for growth and establishment of plants on the forest floor and hence it is not surprising that the diversity of forest floor vegetation should be higher at higher elevations, where the canopy is more open. In a similar way, epiphytes often evidence a mid-elevation peak that correlates with abundant canopy moisture resulting from fog-drip [60]–[62]. At lower elevations epiphytes are often strongly limited by water availability. At higher elevations, as with the woody vegetation, we would expect the diversity of ground-cover vegetation to be constrained by physiological constrains imposed by declining temperature, including effects of frost tolerance, and increasing water stress. Thus, the relationship with elevation may be a product of different limiting environmental factors in the lower and upper parts of the gradient.

Korner [63] concluded that both ecophysiological constraints and the land area per bioclimatic belt are the main factors linked with an altitudinal gradient. Another important factor that interacts with elevation on Rinjani, and on high mountains regions in general, is fire [32]. The occurrence of fire on Rinjani is caused both by intermittent volcanic activity, lightning strikes, and human activities at the higher altitudes. On Rinjani the highest station at 2200 m was dominated by grassland and was clearly exposed to periodic fires. Soot deposits at the base of large trees indicated there was also some history of fire at the 2000 m station, but at lower stations there was no evidence of recent fires. Moreover, the high density of smaller stems at the 2000 m station (Fig. 2) indicates that the site could not have burnt within at least 5 yrs of our census. Dispersal and biotic interactions may also limit species abilities to colonize higher elevations [14], [31], [64]. If pollinators, seed dispersers or seed predators avoid higher elevations or occur there at lower densities this likely to affect plant reproductive success.

### Community composition

One should be careful about interpreting the ecological meaning when comparing studies based on change in community composition, because methods of analysis can strongly influenced the results [65]. To date, variation partition has suggested the total proportion of variation explained by space and environment factors ranges from 16% to 86% for plant species composition in tropical forests [66], [67]. Our multivariate GLMs explained at least 18% of the variance in species composition. Moreover, regardless of the forest strata, elevation explained a substantial proportion of the variance. In agreement with the results for alpha-diversity, forest structure was also an important driver of compositional change for ground-cover plants.

### Above ground biomass

There was no significant relationship between above ground biomass and elevation. However, there was substantial variation among our plots at several elevations suggesting it may be necessary to sample a larger number of plots to obtain a precise estimate of biomass. Nevertheless, Culmsee et al. [28] also did not find any link between above ground biomass and elevation in mountain forests on Sulawesi (Indonesia). In fact, they found a fairly constant above ground biomass along an elevational gradient. Clearly, more work is needed to clarify the underlying causes of the variation in the relationships between above ground biomass and altitude. For example, some researchers have suggested that a hump-shaped relationship may be due to drier climates at lower elevations and the role the Fagaceae family plays in the accumulation of above ground biomass at intermediate elevations in SE Asia [28]. The species-biomass relationship appears to be unimodal in many systems, with diversity peaking at intermediate levels of biomass, especially while comparing sites under the same climate [23]–[25]. However, other patterns have also been found. For instance Waide et al. [20] found 42% of the studies they reviewed, showed no relationship between species diversity and biomass. On Rinjani we also did not find any relationship between above ground biomass and diversity (Fisher's alpha). The factors influencing the biomass and the ways these interact with diversity may be more complex than has often been presumed [28], [53], [68]–[70].

### Community phylogeny structure

Community phylogenetic structure may be random, clustered or overdispersed [42]. On Rinjani, we found assemblages were phylogenetically randomly structured below the 1600 m and significantly clustered above this altitude, under the null hypothesis that assemblages at a particular elevation were a phylogenetically random sample of species drawn from the total species-pool. This indicates that communities at higher elevations (>1600 m) were more closely related to one another than expected by chance. Bryant et al. [71] also found non uniformed phylogeny structure along elevational transect in Colorado. However, they found a tendency to overdispersion toward higher altitudes. Phylogenetic relatedness is often interpreted as a proxy to ecological similarity, because closely related species are likely to be phenotypically similar [72]. Based on this assumption, environmental filtering is predicted to lead to phylogenetic clustering. Thus, our results may be interpreted as suggesting that environmental filtering is stronger at higher elevations on Rinjani. This in turn supports the ecophysiological constraints hypothesis for the pattern of declining diversity with increasing elevation.

We sampled plant communities along an elevational gradient on Mt Rinjani and our study controlled for both sampling regime and sample area. However, it is important to point out that the relationships we report are derived from a single transect located on the north-slope of the mountain. Further sampling from other locations on Rinjani and on other tropical volcanoes will be required to understand the generality of the relationships. The three strata of woody vegetation exhibited decreases in diversity with increasing elevation. Meanwhile, herbaceous vegetation evidenced a peak at mid elevations, which may reflect an interaction between higher canopy cover at lower elevations and physiological constraints at higher elevations. Elevation itself is not an ecological factor affecting plant distribution, but interacts through the effects of local climate on ecological processes [63]. Significant phylogenetic clustering within assemblages at higher evelvations suggests community assembly may be increasingly driven by environmental filtering as elevation increases. Ecophysiological limits may therefore restrict the number of species that can exist at higher elevations on Rinjani. There was no significant relationship between above ground biomass and elevation or diversity.

## Supporting Information

Table S1 **Summary of models for alpha and beta-diversity of different components of vegetation on Mount Rinjani, Indonesia.** Models were arranged according to ΔAIC value. Variables included in the models were elevation, LAI, slope and their respective interactive terms. EL = elevation, SL = slope, LAI = leaf area index.
(DOCX)

Table S2 **Overall species found at Rinjani's plots. G = ground stratum, U = understory stratum, S = subcanopy, and C = canopy.**
(DOCX)

Table S3 **Summary of models examined for above ground biomass of vegetation on Mt Rinjani.** Models are arranged according to ΔAIC value. *K* refers to numbers of parameters included. EL = elevation and SL = slope. We included elevation and slope in the maximal model (all stations 2200 m included).
(DOCX)

Figure S1 **Non- metric multidimensional scaling (NMDS) ordination of ground-cover plant assemblages on Mount Rinjani, Indonesia.** The contours show different elevations and the letters represent different species (the identity of each letter is found in Table S2).
(TIF)

Figure S2 **Non- metric multidimensional scaling (NMDS) ordination of understorey plant assemblages on Mount Rinjani, Indonesia.** The contours show different elevations and the letters represent different species (the identity of each letter is found in Table S2).
(TIF)

Figure S3 **Non- metric multidimensional scaling (NMDS) ordination of subcanopy plant assemblages on Mount Rinjani, Indonesia.** The contours show different elevations and the letters represent different species (the identity of each letter is found in Table S2).
(TIF)

Figure S4 **Non- metric multidimensional scaling (NMDS) ordination of canopy plant assemblages on Mount Rinjani, Indonesia.** The contours show different elevations and the letters represent different species (the identity of each letter is found in Table S2).
(TIF)
