# Supplementary material for: Factors Determining Forest Diversity and Biomass on a Tropical Volcano, Mt. Rinjani, Lombok, Indonesia
Source: PLoS One. 2013 Jul 23;8(7):e67720. doi: 10.1371/journal.pone.0067720 (PMC3720856; doi:10.1371/journal.pone.0067720)
Supplement: Table S1 — Summary of models for alpha and beta-diversity of different components of vegetation on Mount Rinjani, Indonesia. Models were arranged according to ΔAIC value. Variables included in the models were elevation, LAI, slope and their respective interactive terms. EL = elevation, SL = slope, LAI = leaf area index. (DOCX) [file pone.0067720.s001.docx]

**Table S1.** Summary of models for alpha and beta-diversity of different components of vegetation on Mount Rinjani, Indonesia. Models were arranged according to ∆AIC value. Variables included in the models were elevation, LAI, slope and their respective interactive terms. EL=elevation, SL=slope, LAI=leaf area index.

| Type of diversity | Forest stratum | Models |  | k | AIC | ***∆AIC*** |
| --- | --- | --- | --- | --- | --- | --- |
| alpha-diversity | Ground | Y ~EL+EL^2^+LAI |  | 5 | 46.2 | 0.00 |
|  | cover plants | Y~EL+EL^2^+LAI+SL |  | 6 | 49.74 | 3.54 |
|  |  | Y~EL+EL^2^+LAI+LAI:EL+LAI:EL^2^ |  | 7 | 54.67 | 8.47 |
|  | Understory | Y~EL |  | 3 | 81.30 | 0.00 |
|  | plants | Y~EL+LAI |  | 4 | 81.88 | 0.58 |
|  |  | Y~EL+SL |  | 4 | 84.66 | 3.35 |
|  | Subcanopy | Y~EL |  | 3 | 90.14 | 0.00 |
|  | plants | Y~EL+SL |  | 4 | 92.89 | 2.75 |
|  |  | Y~EL+LAI |  | 4 | 93.17 | 3.03 |
|  | Canopy | Y~EL |  | 3 | 89.95 | 0.00 |
|  | plants | Y~EL+SL |  | 4 | 93.15 | 3.20 |
|  |  | Y~EL+LAI |  | 4 | 93.32 | 3.36 |
| beta-diversity | Ground | Y~EL+LAI+SL+EL:LAI+EL:SL |  | 7 | -418.11 | 0.00 |
|  | Cover plants | Y~EL+LAI+SL+EL:LAI+EL:SL+LAI:SL+EL:LAI:SL |  | 9 | -411.52 | 6.59 |
|  |  | Y~EL+LAI+SL+EL:LAI+EL:SL+LAI:SL |  | 8 | -411.52 | 6.59 |
|  | Understory | Y~EL+LAI+SL+EL:LAI+EL:SL |  | 7 | -436.7 | 0.00 |
|  | plants | Y~EL+LAI+SL+EL:LAI+EL:SL+LAI:SL+EL:LAI:SL |  | 9 | -405.16 | 31,54 |
|  |  | Y~EL+LAI+SL+EL:LAI+EL:SL+LAI:SL |  | 8 | -405.16 | 31.54 |
|  | Subcanopy | Y~EL+LAI+SL+EL:LAI+LAI:SL |  | 7 | -415.16 | 0.00 |
|  | plants | Y~EL+LAI+SL+EL:LAI+EL:SL |  | 7 | -415.42 | 0.37 |
|  |  | Y~EL+LAI+SL+EL:LAI+EL:SL+LAI:SL+EL:LAI:SL |  | 9 | -400.29 | 15.51 |
|  | Canopy | Y~EL+LAI+SL+EL:LAI+EL:SL |  | 7 | -415.74 | 0.00 |
|  | plants | Y~EL+LAI+SL+EL:LAI+EL:SL+LAI:SL+EL:LAI:SL |  | 9 | -398.74 | 17.00 |
|  |  | Y~EL+LAI+SL+EL:LAI+EL:SL+LAI:SL |  | 8 | -398.74 | 17.00 |
