# Supplementary material for: Factors Determining Forest Diversity and Biomass on a Tropical Volcano, Mt. Rinjani, Lombok, Indonesia
Source: PLoS One. 2013 Jul 23;8(7):e67720. doi: 10.1371/journal.pone.0067720 (PMC3720856; doi:10.1371/journal.pone.0067720)
Supplement: Table S2 — Overall species found at Rinjani's plots. G = ground stratum, U = understory stratum, S = subcanopy, and C = canopy. (DOCX) [file pone.0067720.s002.docx]

**Table S2**: Overall species found at Rinjani’s plots. G=ground stratum, U=understory stratum, S=subcanopy, and C= canopy

| Species names | Family | G | U | S | C | Code in  NMDS |
| --- | --- | --- | --- | --- | --- | --- |
| *Acer niveum* Blume | Aceraceae | + |  |  |  | A |
| *Actinodaphne glomerata* Nees | Lauraceae |  | + |  |  | B |
| *Adiantum flabellulatum* Wall. | Adiantheceae | + |  |  |  | C |
| *Adina minutiflora* Valeton | Rubiaceae |  |  |  | + | DF |
| *Adinandra javanica* Choisy | Theaceae |  | + | + | + | D |
| *Anaphalis longifolia* DC. | Asteraceae | + |  |  |  | E |
| *Aporosa octandra* (Buch.-Ham.ex D.Don) A.R.Vickery | Euphorbiaceae |  | + |  | + | F |
| *Ardisia crenata* Roxb. | Myrsinaceae |  | + |  |  | H |
| *Ardisia crispa* (Thunb.) A.DC. | Myrsinaceae |  |  | + |  | DG |
| *Ardisia elliptica* Thunb. | Myrsinaceae |  |  | + | + | DH |
| *Ardisia korthalsiana* Scheff. | Myrsinaceae |  | + |  |  | G |
| *Ardisia sp. 1* | Myrsinaceae |  |  | + | + | DI |
| *Asplenium caudatum* G.Forst. | Aspleniaceae | + |  |  |  | I |
| *Asplenium salignum* Blume | Aspleniaceae | + |  |  |  | J |
| *Astronia macrophylla Blume* | Melastomataceae |  |  |  | + | DJ |
| *Basilicum polystachyon* Moench. | Lamiaceae |  | + |  |  | K |
| *Calophyllum soulattri* “Burm.” ex F.Mull. | Clusiaceae |  | + | + | + | L |
| *Canthium dicoccum* (Gaertner) Teijsm.&Binnend | Rubiaceae |  | + |  |  | M |
| *Casuarina junghuhniana* Miq | Casuarinaceae |  | + |  | + | N |
| *Chionanthus sp.* | Oleaceae |  | + |  |  | O |
| *Chloranthus officinalis* Blume. | Chloranthaceae | + |  |  |  | P |
| *Clethra javanica* Turcz. | Clethraceae |  | + | + | + | Q |
| *Cyathea sp.* | Cyatheaceae | + |  |  |  | T |
| *Cynoglossum javanicum*Thunb.ex Lehm*.* | Boraginaceae | + |  |  |  | U |
| *Decaspermum fruticosum* J.R. Forst. & G.Forst | Myrtaceae |  | + |  |  | V |
| *Dendrocnide stimulans* (L.f.)Chew | Urticaceae |  | + |  |  | W |
| *Didymochlaena truncatula* (Sw.) J.Sm. | Dryopteridaceae | + |  |  |  | X |
| *Dodonaea viscosa* Jacq. | Sapindaceae |  | + | + |  | Y |
| *Elatostema sessile* J.R. Forst. & G.Forst | Urticaceae | + |  |  |  | Z |
| *Embelia ribes* Burm.f. | Myrsinaceae |  | + |  |  | AA |
| *Engelhardtia spicata* Blume | Juglandaceae | + | + |  | + | AB |
| *Euonymus javanicus* Blume | Celastraceae |  |  | + | + | DK |
| *Eurya nitida* Korth. | Theaceae |  | + |  | + | AC |
| *Ficus fistulosa* Reinw. Ex Blume | Moraceae |  | + |  |  | AE |
| *Ficus punctata* Thunb. | Moraceae | + |  |  |  | AF |
| *Ficus sp.1* | Moraceae |  |  | + |  | DL |
| *Ficus sp.2* | Moraceae | + | + |  |  | AG |
| *Flacourtia rukam* Zoll. & Moritzi | Flacourtiaceae | + |  |  |  | AH |
| *Geniostoma rupestre* J.R. Forst. & G.Forst | Loganiaceae | + | + |  |  | AI |
| *Glochidion rubrum* Blume | Euphorbiaceae |  |  |  | + | DM |
| *Gomphandra javanica* Valeton | Icacinaceae | + |  |  |  | AJ |
| *Gomphandra sp.* | Icacinaceae |  |  | + | + | DN |
| *Guioa diplopetala* Radlk | Sapindaceae |  | + | + | + | AQ |
| *Homalium caryophyllaceum* Benth. | Flacourtiaceae |  | + | + | + | AR |
| *Ixora cf. grandifolia* | Rubiaceae |  | + | + | + | BF |
| *Laportea stimulans* Miq. | Urticaceae |  |  | + |  | DO |
| *Leucosyke capitellata* Wedd. | Urticaceae |  | + |  |  | BG |
| *Litsea elliptica* Blume | Lauraceae |  |  |  | + | DP |
| *Litsea machilifolia* Gamble | Lauraceae |  | + |  |  | BH |
| *Litsea mappacea* Boerl | Lauraceae |  |  | + | + | DQ |
| *Lycopodium cernuum* L. | Lycopodiaceae | + |  |  |  | BI |
| *Mastixia trichotoma* Blume | Cornaceae |  | + | + |  | BJ |
| *Matricaria sp.* | Asteraceae | + |  |  |  | BK |
| *Melastoma decemfidum* Roxb | Melastomataceae |  | + |  |  | BL |
| *Melastoma malabathricum* L. | Melastomataceae |  | + | + |  | BM |
| *Meliosma pinnata* (Roxb.) Maxim. | Meliosmaceae |  | + |  |  | BN |
| *Microlepia sp.1* | Dennstaedtiaceae | + |  |  |  | BO |
| *Microlepia sp.2* | Dennstaedtiaceae | + |  |  |  | BP |
| *Microlepia sp.3* | Dennstaedtiaceae | + |  |  |  | BQ |
| *Myrica javanica* Blume | Myricaceae |  |  |  | + | DR |
| *Nauclea obtusa* Blume | Rubiaceae |  |  | + | + | DS |
| *Neolitsea cassiaefolia* (Bl.) | Lauraceae |  | + | + |  | BS |
| *Neolitsea javanica* (Blume) Baker | Lauraceae |  | + |  |  | BT |
| *Nephrolepis auriculata* (L.) Trimen | Oleandraceae | + |  |  |  | BU |
| *Petunga microcarpa* DC. | Rubiaceae |  |  | + | + | DT |
| *Podocarpus neriifolius* D.Don | Podocarpaceae |  |  |  | + | DU |
| *Polyosma ilicifolia* Blume | Escalloniaceae |  | + | + |  | BV |
| *Polystichum aculeatum* (L.) Roth | Dryopteridaceae | + |  |  |  | BW |
| *Psychotria laxiflora* Blume | Rubiaceae | + |  |  |  | BX |
| *Pteris biaurita* L. | Pteridaceae | + |  |  |  | BY |
| *Pterospermum javanicum* Jungh | Sterculiaceae |  | + |  |  | BZ |
| *Rapanea hasseltii* Mez | Myrsinaceae |  | + | + |  | CA |
| *Rubus moluccanus* L. | Rosaceae | + |  |  |  | CF |
| *Salacia macrophylla* Blume | Celastraceae |  |  |  | + | DV |
| *Sapium baccatum* Roxb | Euphorbiaceae |  | + | + | + | CH |
| *Saprosma sp.* | Rubiaceae |  | + |  |  | CI |
| *Saurauia sp.* | Actinidiaceae |  |  | + | + | DW |
| *Saurauia umbellata* Koord. & Valeton | Actinidiaceae |  | + |  |  | CJ |
| *Scolopia spinosa* Warb. | Flacourtiaceae |  |  | + | + | DX |
| *Selaginella intermedia* (Bl.) | Selaginellaceae | + |  |  |  | CL |
| *Selaginella plana* (Desv.ex Poir.) Hieron | Selaginellaceae | + |  |  |  | CK |
| *Selliguea feei* Bory | Polypodiaceae | + |  |  |  | CM |
| *Symplocos adenophylla* Wall. & G. Don | Symplocaceae |  | + |  |  | CQ |
| *Symplocos brandisii* Koord. & Valeton | Symplocaceae |  | + | + |  | CR |
| *Syzigium glomeruliferum* Amshoff | Myrtaceae |  | + | + | + | CS |
| *Syzigium sexangulatum* (Miq.) Amshoff | Myrtaceae |  | + | + | + | CT |
| *Syzygium acuminatissimum* DC. | Myrtaceae |  | + | + |  | CU |
| *Syzygium laxiflorum* DC. | Myrtaceae |  |  |  | + | DY |
| *Syzygium polyanthum.*Miq | Myrtaceae |  | + |  |  | CW |
| *Syzygium polycephalum* (Miq.) Merr &L.M. Perry | Myrtaceae |  | + |  |  | CX |
| *Syzygium racemosum* DC. | Myrtaceae | + | + | + | + | CV |
| *Syzygium sp. 2* | Myrtaceae |  |  |  | + | DZ |
| *Syzygium sp. 1* | Myrtaceae |  |  | + | + | EA |
| *Uncaria glabrata* DC*.* | Rubiaceae |  | + |  |  | CY |
| *Viburnum lutescens* Blume | Caprifoliaceae |  | + |  |  | DE |
| *Villebrunea rubescens* Blume | Urticaceae |  |  | + |  | EB |
| *Weinmannia blumei* Planch | Cunoniaceae |  |  | + | + | EC |
| *Unknonw 1* |  | + |  |  |  | R |
| *Unknown 10* |  | + |  |  |  | AS |
| *Unknown 11* |  | + |  |  |  | AT |
| *Unknown 12* |  | + |  |  |  | AU |
| *Unknown 13* |  |  | + |  |  | AV |
| *Unknown 14* |  | + |  |  |  | AW |
| *Unknown 15* |  |  | + |  |  | AX |
| *Unknown 16* |  |  | + |  |  | AY |
| *Unknown 17* |  | + |  |  |  | AZ |
| *Unknown 18* |  | + |  |  |  | BA |
| *Unknown 19* |  |  | + |  |  | BB |
| *Unknown 2* |  | + |  |  |  | S |
| *Unknown 20* |  |  | + |  |  | BC |
| *Unknown 21* |  | + |  |  |  | BD |
| *Unknown 22* |  |  | + |  |  | BE |
| *Unknown 23* |  | + |  |  |  | BR |
| *Unknown 24* |  | + |  |  |  | CB |
| *Unknown 25* |  |  | + |  |  | CC |
| *Unknown 26* |  |  | + |  |  | CD |
| *Unknown 27* |  |  | + |  |  | CE |
| *Unknown 28* |  | + |  |  |  | CG |
| *Unknown 29* |  | + |  |  |  | CN |
| *Unknown 3* |  | + |  |  |  | AD |
| *Unknown 30* |  | + |  |  |  | CO |
| *Unknown 31* |  | + |  |  |  | CP |
| *Unknown 32* |  | + |  |  |  | CZ |
| *Unknown 33* |  | + |  |  |  | DA |
| *Unknown 34* |  | + |  |  |  | DB |
| *Unknown 35* |  | + |  |  |  | DC |
| *Unknown 36* |  | + |  |  |  | DD |
| *Unknown 37* |  |  |  | + |  | ED |
| *Unknown 38* |  |  |  | + | + | EE |
| *Unknown 39* |  |  |  | + | + | EF |
| *Unknown 4* |  | + |  |  |  | AK |
| *Unknown 40* |  |  |  | + | + | EG |
| *Unknown 41* |  |  |  |  | + | EH |
| *Unknown 42* |  |  |  | + |  | EI |
| *Unknown 43* |  |  |  |  | + | EJ |
| *Unknown 44* |  |  |  |  | + | EK |
| *Unknown 45* |  |  |  |  | + | EL |
| *Unknown 46* |  |  |  | + |  | EM |
| *Unknown 47* |  |  |  | + |  | EN |
| *Unknown 5* |  | + |  |  |  | AL |
| *Unknown 6* |  | + |  |  |  | AM |
| *Unknown 7* |  | + |  |  |  | AN |
| *Unknown 8* |  | + |  |  |  | AO |
| *Unknown 9* |  | + |  |  |  | AP |
