# Supplementary material for: Factors Determining Forest Diversity and Biomass on a Tropical Volcano, Mt. Rinjani, Lombok, Indonesia
Source: PLoS One. 2013 Jul 23;8(7):e67720. doi: 10.1371/journal.pone.0067720 (PMC3720856; doi:10.1371/journal.pone.0067720)
Supplement: Table S3 — Summary of models examined for above ground biomass of vegetation on Mt Rinjani. Models are arranged according to ΔAIC value. K refers to numbers of parameters included. EL = elevation and SL = slope. We included elevation and slope in the maximal model (all stations 2200 m included). (DOCX) [file pone.0067720.s003.docx]

**Table S3.** Summary of models examined for above ground biomass of vegetation on Mt Rinjani. Models are arranged according to ∆AIC value. *K* refers to numbers of parameters included. EL=elevation and SL=slope. We included elevation and slope in the maximal model (all stations 2200m included).

| Models | R^2^ | k | AIC | ***∆AIC*** |
| --- | --- | --- | --- | --- |
| y= 18.81-11.35EL-14.32 EL^2^ | 0.35 | 4 | 133.26 | 0 |
| y= 25.33-0.007EL-0.09SL | 0.31 | 4 | 136.47 | 3.21 |
| y= 28.72- 0.006EL | 0.16 | 3 | 138.74 | 5.48 |
