# Supplementary figures and images for: Factors Determining Forest Diversity and Biomass on a Tropical Volcano, Mt. Rinjani, Lombok, Indonesia
Source: PLoS One. 2013 Jul 23;8(7):e67720. doi: 10.1371/journal.pone.0067720 (PMC3720856; doi:10.1371/journal.pone.0067720)

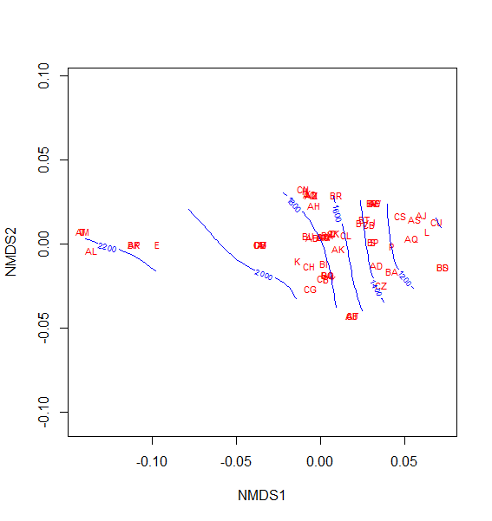

Supplement: Figure S1 — Non- metric multidimensional scaling (NMDS) ordination of ground-cover plant assemblages on Mount Rinjani, Indonesia. The contours show different elevations and the letters represent different species (the identity of each letter is found in Table S2). (TIF) [file pone.0067720.s004.tif]

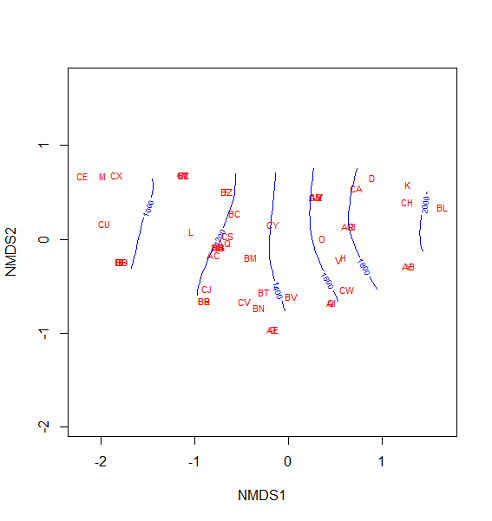

Supplement: Figure S2 — Non- metric multidimensional scaling (NMDS) ordination of understorey plant assemblages on Mount Rinjani, Indonesia. The contours show different elevations and the letters represent different species (the identity of each letter is found in Table S2). (TIF) [file pone.0067720.s005.tif]

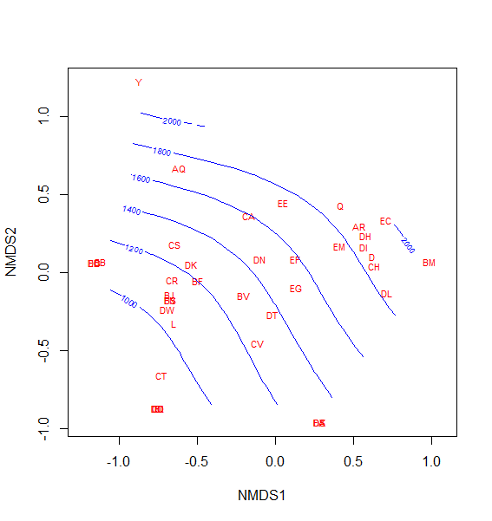

Supplement: Figure S3 — Non- metric multidimensional scaling (NMDS) ordination of subcanopy plant assemblages on Mount Rinjani, Indonesia. The contours show different elevations and the letters represent different species (the identity of each letter is found in Table S2). (TIF) [file pone.0067720.s006.tif]

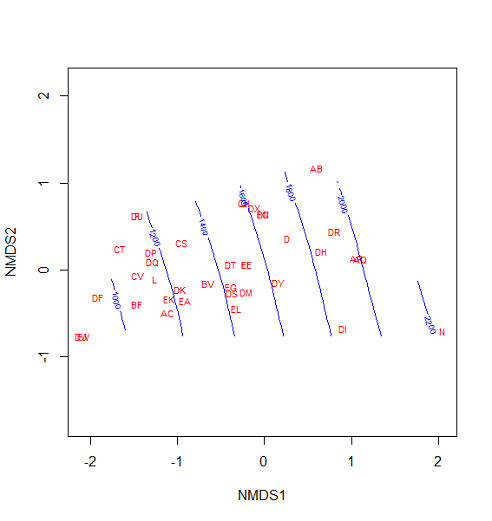

Supplement: Figure S4 — Non- metric multidimensional scaling (NMDS) ordination of canopy plant assemblages on Mount Rinjani, Indonesia. The contours show different elevations and the letters represent different species (the identity of each letter is found in Table S2). (TIF) [file pone.0067720.s007.tif]
